# Referral patterns of Israeli pediatricians of common primary care office procedures

**DOI:** 10.1186/s13584-015-0046-3

**Published:** 2015-12-10

**Authors:** Deena R. Zimmerman, Yona Amitai, Zahi Grossman, Chen Stein-Zamir

**Affiliations:** Jerusalem District Health Office - Ministry of Health, 86 Jaffa Road, Jerusalem, 94341 Israel; Meuchedet Health Services, 31 Yitzchak Mirsky St., Jerusalem, 97284 Israel; Department of Management, Bar Ilan University, Max and Anna Webb St., Ramat Gan, 52900 Israel; Maccabi Health Services, 3 Dov Hausner St., Tel Aviv, 69363 Israel; Braun School of Public Health and Community Medicine Hebrew University in Jerusalem, Ein Karem, PO Box 12272, Jerusalem 91120 Israel

**Keywords:** Children, Primary care, Pediatrics, Office procedures, Referrals, Community, Based medicine

## Abstract

**Background:**

Pediatric primary care is the cornerstone of health care services for children. Performance of common office procedures is an integral part of primary care. Ideally, the community-based primary care pediatrician provides comprehensive health care services and only refers a small minority of patients for consultation. However, knowledge regarding Israeli pediatricians’ practices of office procedures is scant.

**Objectives:**

To describe primary care pediatricians’ patterns in the provision of common office procedures and to analyze factors associated with performance or referral.

**Methods:**

*Design*: Self-completed structured questionnaire consisting of 1) demographic variables; 2) practice characteristics description; 3) List of ten procedures (treatment of subungual hematoma, laceration suturing and adhesive closure, elbow subluxation/reduction , urinary bladder catheterization, supra-pubic aspiration, inguinal hernia reduction, umbilical granuloma and labial fusion treatment, and short lingual frenulum management) followed by questions regarding referral practice for each procedure; and 4) causes and indications for referral when relevant. *Participants*: Primary care pediatricians attending anational pediatric conferences. *Analysis:* Descriptive statistics and association assessment.

**Results:**

The questionnaire was completed by 162 primary care pediatricians, 58.7 % male; mean age 53 ± 9 years, 88.4 % board certified. Of the respondents, 57 % worked in group practices and the remainder solo; salaried employees 68.2 %, independent contractors 31.8 %.

Referral rate varied by procedure; least likely to be referred was labial fusion (7.7 %) and most likely was short lingual frenulum (81.3 %). For most procedures, the most frequent non-performance cause was lack of expertise followed by lack of appropriate conditions. The overall number of procedures in which the response selected was out-of-clinic referral was not associated with demographic or employment characteristics. However, association was found for certain specific procedures (e.g. experience with catheterization, gender with suturing and adhesive closure).

**Conclusions:**

Many common office procedures are referred out of primary care pediatric community settings in Israel. Considerable variability was found among procedures. Lack of experience or lack of appropriate conditions were frequently reported causes for referral and need to be addressed in reducing unnecessary referral with its attendant costs and patient inconvenience. Possible approaches include updates in pediatric residency training, focused in-service training, time allocation and work environment reorganization.

## Background

Pediatric primary care is the cornerstone of health care services for children. [1] The community based primary care pediatrician provides comprehensive health care services and only refers a small minority of patients in their care for further consultation. In a United States based observational study, the subspecialty referral rate was estimated to be 2.3 % children seen [2]. This rate is lower than that found for family practice physicians both the United States (5.1 %) [3]. In an observational study of ten family physicians in Israel, Tabenkin et al. reported a range of referral rates between 7.4-15,9 % [4].

The Organization for Economic Cooperation and Development (OECD) has described Israel’s primary care system as “well developed, accessible and of high quality” [5]. Much of the accessibility can be tied to the National Health Insurance law passed by Israel in 1995. This social welfare law ensures universal access to a comprehensive package of services [6]. Each Israeli resident is free to choose to receive these services from among four health funds [7]. Children under the age of 18 years are entitled to health insurance regardless of residence status [8]. Primary care for Israel’s over 2.5 million children [9] is provided in either in clinics run directly by the health funds or in “independent practices” whose services are contracted by the the health funds. Primary care pediatrics is provided by a combination of pediatricians and family practitioners with younger children more likely to preferentially be seen by pediatricians [10, 11].

Part of primary care is the performance of procedures and the treatment of minor trauma in the office setting. Provision of such services in the office setting has been shown to be cost effective, convenient for patients and professionally satisfying for physicians. It also reduces fragmentation of care and patient waiting times [12]. However, there is world-wide evidence that many primary care physicians are not providing these services in their practices. A study of biopsies and joint injections in Canada found performance rates of under 50 % for three of the four procedure examined [13]. A study of joint injections in Saudi Arabia found less than 10 % performance of almost all treatment [14]. In southern Israel, Menahem et al. found that 65 % of the primary care physicians perform any minor surgical procedures and 46 % perform any muscular injections [15]. In all of the studies, a repeated thread in reasons for not performing the procedures was lack of up to date skills and lack of time.

Primary care provision to children varies between countries. In many European countries [9, 10] and the United States, as well as Israel, pediatricians provide much of the primary care of children. Furthermore, pediatric office procedures differ from their adult counterparts. Therefore, it is important that the extent of office procedure performance by pediatricians be studied. While specialist referral patterns of community based pediatricians have been studied [2], office procedures were not studied in depth. Therefore, the focus of this study is the procedure based treatments that can be done in an office setting but that if not done will require referral for performance in other settings.

The aim of this study is to describe the referral patterns regarding ten common office procedures and analyze the reasons for performance or non-performance.

## Methods

The study was conducted using a structured self-completed anonymous questionnaire. The questionnaire was administered to primary care pediatricians attending one of two national meetings. One meeting was that of the Israeli Ambulatory Pediatric Association, the organization of community based primary care pediatricians. The other was a national meeting of pediatricians sponsored by one of the four health funds. Care was taken to assure no questionnaire duplication. While this was essentially a convenience sample, these meetings attract community based pediatricians and thus the study group was considered to be a representative sample. Furthermore, the demographic and education characteristics were compared to the national surveys of the medical workforce in Israel [16, 17]. The questionnaire contained four main topics 1) Demographic characteristics 2) Professional training 3) Working environment 4) Performance of ten procedures – half derived from the residency curriculum of the Academic Pediatric Association [18] and half from their own clinical experience. These procedures were treatment of subungual hematoma, suturing and adhesive closure of lacerations, reduction of radial head subluxation (“pulled elbow”), urinary bladder catheterization, supra-pubic aspiration, inguinal hernia reduction, umbilical granuloma and labial fusion management, and management of short lingual frenulum that is interfering with successful breastfeeding. For three situations (radial head subluxation, umbilical granuloma and labial fusions), physicians were asked to estimate the annual frequency of presentation of the specific condition.

The response options for the pediatricians’ replies were principally divided into two main categories regarding procedures: treatment within the primary care clinic or referral out to subspecialist or emergency department care. Referral to the nurse within the clinic was considered as treatment in the clinic. When the respondent chose the answer “referral”, several additional questions on causes for referral were included in the questionnaire.

Association with Referrals: We composed a new variable- a score for each individual pediatrician summarizing the overall number of procedures in which the response selected was referral out of the clinic.

Data were analyzed using the Statistical Package for the Social Sciences, SPSS® software 21. The Odds Ratio (OR) and the 95 % confidence intervals (95 % CI) are reported. Continuous variables were compared by the Student t test; dichotomous variables were analyzed by the Pearson chi-square test. A *p*-value < 0.05 was considered significant for all comparisons. Since the study did not involve personal data on patients and the participating pediatricians filled the questionnaires anonymously, ethics committee approval was not needed.

## Results

*Study Population*: The questionnaire was completed by 162 pediatricians. The compliance rate was 70 % of those asked to participate. The general characteristics of the study participants are presented in Table 1. The distribution of pediatricians' according to age groups was: under 44 years (23.5 %), 45–54 years (31.4 %), 55–64 years (34.6 %) and 65 years and above (10.5 %).Of note, the majority (68.2 %) worked for one or more of the health funds as salaried employees with the remainder working as independent contractors. Over half (57 %) worked in group practices. An additional 7 % worked in a specialized form of group practice known as a Child Health Center.

**Table 1:** Demographic and occupational characteristics of respondents

Variable	*N* (%)
Age (mean ± SD)	52.8 ± 9
Age (median)	53.0
Age (range)	31-78 years
Male gender	91/155 (58.7 %)
Professional experience in years (mean ± SD)	22.2 ± 10.2
Professional experience in years (range)	2 - 43 years
Professional experience in years (median)	22 years
Country of Medical Studies: Israel	92/148 (62.2 %)
Former Soviet Union	36/148 (24.3 %)
other	20/148 (13.5 %)
Residency training in Israel	125/136 (95.4 %)
Board certification	137/155 (88.4 %)
Financial Arrangement : Salaried employee	107/157 (68.2 %)
Independent contractors	50/157 (31.8 %)
Practice type: Group practice	81/ 142 (57 %)
Solo practice	50/142 (35.2 %)
Child Health Center	11/142 (7.7 %)
Number of children seen per week (mean ± SD)	188.8 ± 110.9
Number of children seen per week (median)	200
Number of children seen per week (range)	25-500

*Procedures, Referral Rates and Reasons*: The ten procedures studied are presented in Table 2 including the number of respondents, the distribution of answers and the percentage of respondents per answer. Those procedures with an asterix indicate that they appear in the curriculum of the Academic Pediatric Association. There were no differences in the main findings between those procedures that were part of the curriculum and those that were not.

**Table 2:** List of procedures and distribution of physician’s answers

Procedure	Answer 1 *N* (%)	Answer 2 *N* (%)	Answer 3 *N* (%)
Labial fusion (*n* = 156)	Give prescription for estrogen 127 (81.4 %)	Refer to gynecologist 12 (7.7 %)	Separate in the Office 17 (10.9 %)
Subungual hematoma (*n* = 159)	Drain in office 63 (39.6 %)	Do not treat at all 81 (51 %)	Other procedure in office 15 (9.4 %)
*Laceration: adhesive (*n* = 162)	Use tissue adhesives in office 116 (71.6 %)	Refer to ED 20 (12.3 %)	Refer to nurse 26 (16 %)
*Pulled Elbow (*n* = 162)	Reduce in Office 128 (79 %)	Send to ED 33 (20.4 %)	Send for an x ray 1 (0.6 %)
*Urinary catheterization (*n* = 161)	Perform in office 71 (44.1 %)	Refer to expert 61 (37.9 %)	Refer to nurse 29 (18 %)
Umbilical Granuloma (*n* = 158)	Cauterize in Office 56 (35.4 %)	Refer to surgeon 61 (38.6 %)	Follow only 41 (25.9 %)
Inguinal hernia (*n* = 161)	Reduce in office 92 (57.1 %)	Refer to surgeon 69 (42.9 %)	
*Supra-pubic aspiration (*n* = 159)	Perform in office 66 (41.5 %)	Refer to ED 93 (58.5 %)	
*Laceration: suture (*n* = 161)	Suture in Office 46 (28.6 %)	Refer to ED 115 (71.4 %)	
Short frenulum interfering with breastfeeding (*n* = 160)	Incise in Office 12 (7.5 %)	Refer to surgeon 130 (81.3 %)	Recheck age 1 year 18 (11.3 %)

**Fig. 1:**
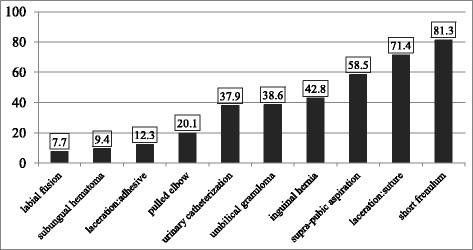
Distribution of referral out of the clinic by procedure

**Fig. 2:**
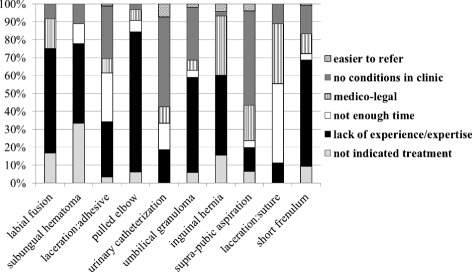
Distribution of reasons for out of the clinic referral by procedure

The referral percentage for each procedure is presented in ascending order in Fig. 1. Least likely to be referred was labial fusion and most likely was short lingual frenulum. Figure 2 shows the distribution of causes given for referral by procedures. For most procedures, the most frequent cause given for non-performance was lack of expertise. The next most common reason was lack of appropriate conditions. The annual presentation of radial head subluxation, umbilical granuloma and labial fusion were similar with approximately 50 respondents stating they saw 1–3 times year, 50 statin 4–6 times per year, 30 stating 7–10 times per year and 25 stating more than 10 times per year.

*Association with Referrals*: The variable reflecting the overall number of procedures referred by each pediatrician was not found to be associated with demographic and employment characteristics including the pediatrician's age, gender, years of professional experience and type of practice. However, in specific procedures a significant association was in fact found:

1. Treating umbilical granuloma (*p* = 0.04) and incision of short frenulum (*p* = 0.03) – the greater the years of paediatrician's professional experience, the less likelihood of referral.
2. Urinary tract infection (UTI) work-up: Pediatricians with less years of professional experience tended to perform the procedure themselves (*p* = 0.002). Pediatricians working in solo practice were more likely to refer children for catheterization compared to those working in HMO clinics and Child Health Centers (OR = 3.19; 95 % CI = 1.5-6.8; *p* = 0.001). A similar trend was found as to referral for supra- pubic aspiration. (OR = 2.78; 95 % CI = 1.24-6.5; *p* = 0.005).
3. Pediatricians who were educated outside Israel tended to refer children for treatment of pulled elbow more frequently than those who studied in Israel. (OR = 2.24; 95 % CI = 0.94-5.35; *p* = 0.036).
4. Female pediatricians tended to refer children for treatment of lacerations (either for suturing or adhesive closure) more often than male pediatricians. (OR = 3.41; 95 % CI = 1.42 – 8.82; *p* = 0.02).
5. Availability of medical equipment in the clinic – pediatricians who worked in clinics with medical equipment (pulse oximeter and nebulizer) were less likely to refer children for UTI work-up (OR = 0.27; 95 % CI = 0.12 – 0.62; *p* = 0.001) and for subungual hematoma. The availability of any medical equipment was highly correlated with the type of clinic, particularly Child Health Centers.

## Discussion

Performance of minor procedures in the office setting has been shown to have numerous advantages. These include cost effectiveness, patient convenience, reduced care fragmentation and waiting times. It has also been shown to provide physicians with professional satisfaction [12]. Nevertheless, studies among family practice physicians have shown that often this performance does not take place [13–15].

The general characteristics of the study participants were compared to those concerning pediatricians which were published in Israel’s Ministry of Health (MOH) report on health professionals in Israel 2013 [16]. Comparison of age groups distribution (55 and 52 % under 55 years in study participants vs. MOH report), gender (55 % vs. 53 % males under 65 years) and country of medical studies (Israel, 62 % vs. 58 %) there was high similarity between the study participants and pediatric professionals in general.

Our main finding is that a fair number of situations that could theoretically be treated in the primary care clinic setting are referred elsewhere for treatment by Israeli community-based primary care pediatricians. This is similar to the study of Sempowski et al. who conducted a mail survey of 108 Canadian family physicians (actual responses 79) regarding four office procedures (dermatological excision, endometrial biopsy, injection of the shoulder and knee). They found performance rates of under 50 % for the latter three and 63 % for skin excision [11] Looking at specific surgical procedures in the southern Israel study, the rates ranged from suturing 6 % for excision to 48 % for of skin lesions and thrombosed hemorrhoids [13]. That range was even greater in our study - from 7.7 % for labial fusion to 81.3 % for short lingual frenulum.

The most common reason for non-performance in our study was the most frequent non-performance cause was lack of expertise followed by lack of appropriate conditions. The finding of lack of expertise is particularly striking in light of the years of experience (mean 22 +/− 9 years) of the respondents in our study. These two reasons were also the most frequent explanations in all the family physicians studies cited.

For most of the procedures studied, lack of expertise cannot be attributed to lack of ongoing exposure to the condition. As seen by common experience, lacerations are extremely common in young children. Search for the possibility of a urinary tract infection is a standard part of the work up for fever in children and by guidelines. Urine cultures in non- toilet trained children must be done by bladder catheterization or aspiration [19]. Most of the study sample estimated that they saw a child with radial head subluxation, umbilical granuloma or labial fusion at least every 2 months on average.

Lack of expertise is clearly the only reason that can be given for not performing reduction of elbow subluxation as it needs no equipment and takes a few seconds to perform. For other procedures, the issue may be less one of ability but rather lack of the appropriate surroundings for performance. This is likely the issue for obtaining a urine culture sample by catheterization or aspiration. This fits well with the findings that pediatricians working in as independent contractors were more likely to refer for UTI work up than those working as salaried employees in health fund clinics. As independent contractors are paid per patient seen and salaried employees are paid by the hour, procedure performance time is of greater financial importance to independent contractors. The difference in willingness to perform procedures based on method of remuneration was also found in Canada. Physicians working in Family Health Networks (paid on a capitation basis) were less likely to cite lack of time as a reason for not performing procedure than physicians who worked on a fee -for -service basis. In the southern Israel study, the issue of lack of remuneration for performing the procedure was cited by 24 % of the respondents.

An alternate explanation for the difference in referral rates based on practice setting is that physicians working as independent contractors are more likely to work without ancillary staff that can assist in the performance of procedures. This is in concert with the finding that pediatricians in solo practice were more likely to refer children for catheterization compared to those in a Child Health Center. This latter likely reflects the greater availability of additional health care workers available in Child Health Centers. The importance of the work environment is demonstrated by the reverse association found between equipment found in the clinic and referral, even if the equipment examined (pulse oximeter, nebulizers) were not needed for any of the procedures examined.

An additional environmental concern is the time needed for a procedure. In children who may need additional enticements to be cooperative, this can be a particular concern. While we do not have the data to calculate physician patient load, the average weekly number of patients in this sample was 200. Further studies should explore this in greater detail in light of the exploratory study of Kushnir et al. showing an association between burnout and referral [20] and Kushnir and Cohen showing growing burnout among Israeli pediatricians [21].

Our score summarizing each individual pediatrician's referral out of the clinic was not found to be associated with demographic variables. There was, however, an association of laceration management with gender in that female pediatricians were more likely to refer than either suture or use tissue adhesives. This is similar to the findings of in a study by Westmore et al. among Canadian family physicians where the authors did not find associations with demographic variables or other than female physicians working outside of the study city (London, Ontario) were most likely to perform procedures themselves. It is also similar to the findings among southern Israel family physicians where male physicians were significantly more likely to perform minor surgical procedures and musculoskeletal injections (OR 2.12 and 2.86, respectively) and female physicians were more likely to refer.

The lower referral rate for UTI workup by catheterization or supra pubic aspiration by pediatricians with less years of experience might be their greater manual/technical skills, as these physician are closer in time to residency training.

### Strengths and limitation

The strength of the study is the completion of the survey by a large number of community-based pediatricians with a nation-wide distribution. The study population (*n* = 162) who completed the questionnaire represent approximately 18 % of Israel’s community-based pediatricians [16]. This is the first study of pediatric referral that focuses on procedures and it includes evaluation of the reasons for the referral.

The main limitation of the study is that it represents self -report of what the pediatrician would do rather than direct observation of what he/she does do. Future studies that involve larger samples of physicians (both pediatricians and family physicians) and applying either direct observation or documented information on referral practice would be valuable.

Future studies would also benefit from the inclusion of additional demographic variable such as number of hours worked per week and geographic location of practice. Additional directions for future research could include questions regarding fear of liability or estimated procedure complication rate.

## Conclusions

In conclusion, many common office procedures are referred out of primary care pediatric community settings. Lack of experience or lack of appropriate conditions are frequent reasons for referral. The implications of these findings are three fold. First, residency training programs should assure that their graduates are proficient in common office procedures such as those listed. To reach this goal, it is important that there is adequate exposure in residency to community based centers. Furthermore, a set curriculum that includes procedures such as composed by Academic Pediatric Association [18] should be developed for Israel and implemented. Second, health funds should provide workshops on these procedures for those who already graduated. Third, health funds should address practice surroundings to assure that the manpower, equipment and time is in place for their performance. As stated by Bitterman and Vinker in their commentary regarding family medicine "For the healthcare system the “extra effort” and investment needed for performance of minor surgical procedures in primary care clinics is small. This rather limited managerial extra effort is very well aligned with economic incentives that emphasize a shift of activities to community and primary care settings as well as with modern consumerism that highlights close to home, readily available services" [22], These issues need to be addressed for pediatrics as well so pediatric primary care can reach its goal in reducing unnecessary patient referral and improving the quality of community-based pediatric health care services.

## References

[1] Cooley WC, McAllister JW, Sherrieb K, Kuhlthau K (2009). Improved outcomes associated with medical home implementation in pediatric primary care. Pediatrics.

[2] Forrest CB, Glade GB, Baker AE (1999). The pediatric primary-specialty care interface: how pediatricians refer children and adolescents to specialty care. Arch Pediatr Adolesc Med.

[3] Forrest CB, Nutting PA, Starfield B, Von Schrader S (2002). Family physicians’ referral decisions results from the ASPN referral study. J Fam Pract.

[4] Tabenkin H, Oren B, Steinmetz C (1998). Referral of patients by family physicians to consultants: a survey of the Israeli Family Practice Research Network. Fam Pract.

[5] OECD Reviews of Health Care Quality: Israel 2012 - Raising Standards. OECD Publishing. Available on line at http://www.keepeek.com/Digital-Asset-Management/oecd/social-issues-migration-health/oecd-reviews-of-health-care-quality-israel-2012_9789264029941-en#page1. Accessed Aug 7,2014.

[6] State of Israel, Ministry of Health. Rights of the insured under the national health insurance law (1995). http://www.health.gov.il/English/Topics/RightsInsured/RightsUnderLaw/Pages/default.aspx. Accessed Aug 7,1014.

[7] Rosen B (2009). Israel: health system review. Health Syst Transit.

[8] http://www.kolzchut.org.il/en/Health_Insurance_for_Non-Resident_Children_in_Israel. Accessed Aug 7,2014.

[9] http://mfa.gov.il/MFA/AboutIsrael/People/Pages/Israeli_children_statistics_2011.aspx Accessed Aug 7, 2014.

[10] Katz M, Rubino A, Collier J, Rosen J, Ehrich JHH (2002). Demography of pediatric primary care in Europe:delivery of care and training. Pediatr.

[11] van Esso E, del Torso S, Hadjipanayis A, Biver A, Jaeger-Roman E, Wtterfrn B (2010). Paediatric primary care in Europe: variation between countries. Arch Dis Child.

[12] Westmore SJ, Agbayani R, Bass MJ (1998). Procedures in ambulatory care: which family physicians do what in southwestern Ontario?. Can Fam Physician.

[13] Sempowski IP, Rungi AA, Seguin R (2006). A cross sectional survey of urban Canadian family physicians’ provision of minor office procedures. BMC Fam Pract.

[14] Al-Ahaideb A, Khoshhal K, Aksiddiky A, Heissam K, Alzakari A, Alsaleh K (2012). Patterne and obstacles of provision of minor orthpopedic procedures among primary care physicians in Saudia Arabia. Int J Health Sci (Qassim).

[15] Menahem S, Nazarenko A, Shvartzman P (2014). Minor surgical procedures and musculoskeletal injections by primary care physicians – an Israeli experience. Isr J Health Policy Res.

[16] Manpower in the Health Professions in Israel in 2013; Ministry of Health report, Jerusalem Israel 2014. Available at: http://www.health.gov.il/PublicationsFIles/manpower2013.pdf

[17] Haklai Z, Applbaum Y, Tal O, Aburbeh M, Goldberger MF (2013). Female physician; trends and likely impacts on health care in Israel. Isr J Health Policy Res.

[18] Szumlas GA (2002). Development of an office-based curriculum of common pediatric primary care skills for residents. Acad Med.

[19] American Academy of Pediatrics (2011). Urinary tract infection: clinical practice guideline for the diagnosis and management of the initial UTI in febrile infants and children 2 to 24 month. Pediatrics.

[20] Kushnir T, Greenberg D, Madjar N, Hadari I, Yermiahu Y, Bachner YG (2014). Is burnout associated with referral rates among primary care physicians in community clinics?. Fam Pract.

[21] Kushnir T, Cohen AH (2006). Job structure and burnout among primary care pediatricians. Work.

[22] Bitterman H, Vinker S (2014). Extending the boundaries of family medicine to perform manual procedures. Isr J Health Policy Res.

